# Genetic variation in the mitochondrial 16S ribosomal RNA gene of *Ixodes scapularis* (Acari: Ixodidae)

**DOI:** 10.1186/s13071-014-0530-6

**Published:** 2014-11-28

**Authors:** Chantel N Krakowetz, L Robbin Lindsay, Neil B Chilton

**Affiliations:** Department of Biology, University of Saskatchewan, Saskatoon, SK S7N 5E2 Canada; Public Health Agency of Canada, National Microbiology Laboratory, Winnipeg, MB R3E 3R2 Canada

**Keywords:** *Ixodes scapularis*, Genetic variation, Mitochondrial 16S rRNA gene, Secondary structure, Population genetics, Phylogeography

## Abstract

**Background:**

*Ixodes scapularis* is a vector of several human pathogens in the United States, and there is geographical variation in the relative number of persons infected with these pathogens. Geographically isolated populations of *I. scapularis* have established or are in the process of establishing in southern Canada. Knowledge of the genetic variation within and among these populations may provide insight into their geographical origins in the United States and the potential risk of exposure of Canadians to the different pathogens carried by *I. scapularis*.

**Methods:**

Part of the mitochondrial (mt) 16S ribosomal (r) RNA gene was amplified by PCR from 582 ticks collected from southern Canada, and Minnesota and Rhode Island in the United States. Sequence variation was examined in relation to the predicted secondary structure of the gene. Genetic diversity among populations was also determined.

**Results:**

DNA sequence analyses revealed 52 haplotypes. Most mutational alterations in DNA sequence occurred at unpaired sites or represented partial compensatory base pair changes that maintained the stability of the secondary structure. Significant genetic variation was detected within and among populations in different geographical regions. A greater proportion of the haplotypes of *I. scapularis* from the Canadian Prairie Provinces were found in the Midwest of the United States than in other regions, whereas more of the haplotypes of *I. scapularis* from the Canadian Central and Atlantic Provinces occurred in the Northeast of the United States. Nonetheless, 58% of *I. scapularis* were of a haplotype that occurs in the Midwest and Northeast of the United States; thus, their geographical origins could not be determined.

**Conclusions:**

There is considerable genetic variation in the mt 16S rRNA gene of *I. scapularis*. There is some evidence to support the hypothesis that some lineages of *I. scapularis* in the Atlantic and Central Provinces of Canada may be derived from colonizing individuals originating in the Northeast of the United States, whereas those in the Prairie Provinces may be derived from individuals originating in the Midwest of the United States. However, additional genetic markers are needed to test hypotheses concerning the geographical origins of *I. scapularis* in Canada.

**Electronic supplementary material:**

The online version of this article (doi:10.1186/s13071-014-0530-6) contains supplementary material, which is available to authorized users.

## Background

Ticks are important vectors of human and animal pathogens [1,2]. The incidences of tick-borne diseases are increasing [1-3], due in part, to the expansion of the distribution of some tick species into new geographical areas [2]. Over the past 10 years, the distribution of the blacklegged tick (*Ixode*s *scapularis*) in North America has continued to expand in the Upper Midwest of the United States [4,5], and in southern Canada [6-10]. This range expansion is important from a public health perspective because *I. scapularis* is the principal vector of *Borrelia burgdorferi sensu stricto*, the causative agent of Lyme disease in North America [11]. Blacklegged ticks are also important vectors of the etiologic agents of human granulocytic anaplasmosis (*Anaplasma phagocytophilum*) [12], human and rodent babesiosis (*Babesia microti*) [13], and tick-borne encephalitis (Powassan virus) [14]. The relative occurrences of these diseases, and the prevalences of the different strains of pathogens, vary throughout the distributional range of *I. scapularis* [3,14-18]. Understanding the evolutionary ecology of these vector-borne diseases requires detailed knowledge of the biology, ecology, and population genetics of the vector and the pathogens it carries [19].

The distribution of *I. scapularis* in the United States is divided into three geographically isolated foci: the Northeast, Midwest, and the South [20-23]. There are also geographically isolated populations of *I. scapularis* in several provinces in southern Canada. The first of these Canadian populations established at the Long Point peninsula, which includes the Long Point Provincial Park and adjacent National Wildlife area (Ontario) was described in the early 1970s [24], whereas the next two populations, at Point Peele National Park and Rondeau Provincial Park (Ontario), did not establish until the 1990s [25,26]. Other populations have now become established or are in the process of establishing in Ontario, Nova Scotia, New Brunswick, Manitoba and Quebec [7,9,10,27,28]. It has been proposed that migratory passerines are transporting large numbers of *I. scapularis* larvae and nymphs into Canada from the United States each year during their spring migration [7,29].

Studies have shown there are two major lineages or clades of *I. scapularis* in the United States [30-33]. Individuals of the Southern clade have only been reported from North Carolina, South Carolina, Georgia, Oklahoma, Texas, Arkansas, and Florida, whereas those of the American clade occur primarily in the Northeast and Midwest, but also occur in some southern states [18,30-33]. Blacklegged ticks in southern Canada were also shown to belong to the American clade based on analyses of the DNA sequences of part of Domains IV and V of the mitochondrial (mt) 16S ribosomal RNA (rRNA) gene [34]. The absence of individuals of the Southern clade, combined with significant differences in genetic structure among six established populations, suggested that *I. scapularis* populations in southern Canada were founded by colonizing individuals that originated from different populations in the Northeast and Midwest of the United States [34]. In addition, eight of the 19 haplotypes detected among *I. scapularis* in southern Canada had not been reported previously in studies conducted in the United States. These eight haplotypes also represented 27% of all ticks characterized in southern Canada [34]. This suggested that the extent of the genetic diversity in *I. scapularis*, based on the DNA sequences of Domains IV and V of the mt 16S rRNA gene, was more extensive than previously thought*.* Therefore, in the present study, we assessed the extent of the variation in the DNA sequences of the mt 16S rRNA gene for *I. scapularis*, both within and among populations in southern Canada, and the Midwest and Northeast of the United States. We also examined the phylogeographical relationships of *I. scapularis* to determine the possible geographical origins of the different tick populations in southern Canada.

## Methods

### Tick samples

**Figure 1:**
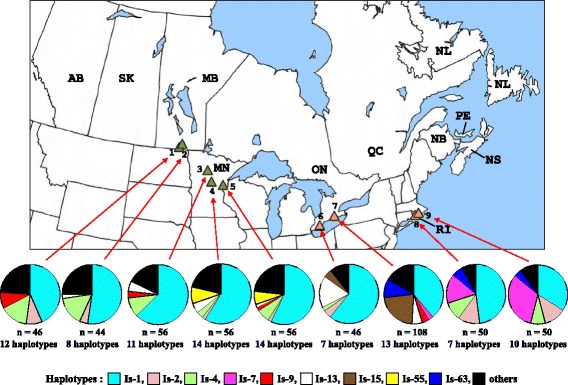
**Established populations in Canada and the United States from where** ***I. scapularis*** **were collected, and the relative abundance of different 16S haplotypes within each population.** Green triangles represent populations in the western geographical region: 1) Pembina Valley Provincial Park, 2) Stanley Trail, 3) Itasca State Park, 4) Camp Ripley, and 5) St. Croix State Park, while orange triangles tick populations in the eastern geographical region: 6) Point Pelee National Park, 7) Long Point Provincial Park, 8) Trustom Pond, South Kingstown, and 9) Hazard Island, South Kingstown.

A total of 582 *I. scapularis* were collected between 2000 and 2011 (Table 1 and Additional file 1: Table S1). Of these, 70 were adventitious (i.e., ticks not associated with known resident populations) and were collected from hosts or the environment in different Canadian provinces, while 512 ticks were collected by drag sampling [35] at nine localities in Canada and the United States, each of which represented an established population of *I. scapularis* (Figure 1). For some data analyses, the collection localities of all ticks were grouped into one of two geographical regions. The “western” region comprised the Canadian Prairie Provinces (Alberta, Saskatchewan and Manitoba) and Minnesota in the Midwest of the United States. The “eastern” region included the Central Provinces (Ontario and Quebec) and Atlantic Provinces (Newfoundland, Nova Scotia, Prince Edward Island and New Brunswick) of Canada, and Rhode Island in the Northeast of the United States.

**Table 1:** **The number of** ***I. scapularis*** **collected between 2000 and 2011 from different regions of North America**

**Region**	**No. of adventitious ticks**	**No. of ticks from established populations**	**Total**
**Canada**			
Prairie Provinces			
Alberta (AB)	2	0	2
Saskatchewan (SK)	6	0	6
Manitoba (MB)	6	90	96
Central Provinces			
Ontario (ON)	12	154	166
Quebec (QC)	22	0	22
Atlantic Provinces			
New Brunswick (NB)	6	0	6
Prince Edward Island (PE)	3	0	3
Nova Scotia (NS)	11	0	11
Newfoundland (NL)	2	0	2
United States			
Midwest			
Minnesota (MN)	0	168	168
Northeast			
Rhode Island (RI)	0	100	100
Total	70	512	582

All ticks were adults, except for those from Rhode Island which were questing nymphs.

The locations of the established populations are shown in Figure 1.

### DNA extraction and PCR amplification

The total genomic (g) DNA of each tick was extracted using the QIAamp DNA Mini Kit™ or the DNeasy Blood & Tissue Kit™ (Qiagen), as described previously [6,36]. A region (~400 bp) spanning Domains IV and V of the mt 16S rRNA gene was amplified by PCR from the total gDNA of each tick using the primers 16S − 1 (5’-CTGCTCAATGATTTTTTAAATTGCTGTGG -3’) and 16S + 1 (5’-CCGGTCTGAACTCAGATCAAGT-3’) [31]. PCRs were performed in reaction mixtures (25 μl or 50 μl) containing PCR buffer with KCl, 1.75 mM MgCl_2_, 200 μM of each dNTP, 25 ρmol of each primer, 0.5-1.25 U of *Taq* polymerase, and 1–2 μl of gDNA template. The cycling conditions used were 96°C for 5 min, then 30 cycles of 94°C for 30 s, 52°C for 30 s and 72°C for 30 s, and a final extension at 72°C for 5 min. All PCR products were examined on 1.5% agarose-TBE gels to verify that each amplicon represented a single band of ~450 bp.

### Single-strand conformation polymorphism (SSCP) and DNA sequencing

All amplicons were subjected to single-strand conformation polymorphism (SSCP) analysis, a mutation-scanning technique that is highly effective in displaying genetic variation among amplicons (150–450 bp) that differ in DNA sequence by one or more nucleotides [37]. The SSCP methodology used followed that described previously [34], except that 0–4.5 μl of DNase-free water and 5 μl of loading buffer (Gel Tracking Dye™, Promega) were added to 0.5-5 μl of each amplicon. Where possible, multiple amplicons of each SSCP profile type were subjected to automated sequencing using primers 16S − 1 and 16S + 1 in separate reactions. Amplicons were purified prior to sequencing. This was achieved by adding 1 μl of a mixture containing 3 U of exonuclease I, 0.15 U of shrimp alkaline phosphatase, and 0.7 μl of 1X PCR buffer to 10 μl of each amplicon, and incubating the samples at 37°C for 15 min. Subsequently, increasing the temperature to 80°C for 15 min inactivated the enzymes. The sequences of the haplotypes have been deposited in GenBank™ under the accession numbers HG916768-HG916804. The numerical system for haplotype designation used herein follows that of Krakowetz *et al*. [34]. BLAST searches (GenBank) were performed on the sequence data obtained to determine if the haplotypes detected in the present study were identical to those in other studies, but where different haplotype designations are used (see Additional file 2: Table S2).

### Sequence alignment, secondary structure and phylogenetic analyses

Sequences were aligned manually, but then modified according to the predicted secondary structure of Domains IV and V of the mt 16S rRNA gene that was constructed for *I. scapularis* based on the models of other organisms [38]. Phylogenetic analyses using the neighbor-joining (NJ) method were carried out using PAUP v4.0b10 [39]. The DNA sequences of the mt 16S rRNA gene of *I. pacificus* (GenBank accession no. AF309008) and several haplotypes of the Southern and American clades of *I. scapularis* (see Supplemental Table S2 for accession nos.) [31,32] were included in the analyses. A bootstrap analysis (1000 replicates) was used to determine the relative support for groups in the NJ tree.

### Population genetics and phylogeographical analyses

The haplotype (*h*) and nucleotide (π) diversities of *I. scapularis* within established populations were determined using Arlequin [40]. Only data from the established populations of *I. scapularis* were included in the analyses. Tests for selective neutrality, Tajima’s *D* [41] and Fu’s *F*_*S*_ [42], were also performed using Arlequin. Under the neutral model, *D* and *F*_*S*_ values should be approximately zero. Significantly negative *D* (*p* <0.05) and *F*_*S*_ (*p* <0.02) values are indicative of populations undergoing expansion, whereas significantly positive values are characteristic of populations undergoing bottlenecks [43]. Arlequin was also used to conduct a Chakraborty’s test [44], which determines if there were significantly more haplotypes in a population than expected under neutrality, and to calculate a measure (pairwise *F*_*ST*_) of genetic differentiation between each pair of populations. The significance of departures of *F*_*ST*_ values from zero was tested using 1000 permutations. A hierarchical analysis of molecular variance (AMOVA) was conducted using Arlequin to determine if there was genetic structuring within and among populations in different geographical regions. For this analysis, the nine established populations of *I. scapularis* were divided into four groups based on the province (Canada) or state (United States) in which they were located. A Mantel test was also conducted using Arlequin (1000 permutations) to determine if there was a correlation between genetic (*F*_*ST*_) and geographical (km) distances among populations.

Rarefaction curves were generated using EstimateS [45] to estimate the total number of haplotypes that can be expected in a sample (i.e., based on the asymptote of the curve), and the extent to which the majority of haplotypes have been sampled. These analyses were performed for the populations in the western and eastern geographical regions, and the pooled population data of *I. scapularis*. EstimateS (1000 runs) was also used to determine Chao 2 values, estimators of the expected number of haplotypes in a sample [46].

A minimum spanning network tree depicting the relationships of the haplotypes was produced using TCS version 1.21 [47]. This analysis also included other haplotypes of the American clade from Canada [34], the Northeast (e.g., Pennsylvania, Connecticut, New York, New Jersey, Massachusetts, Maryland and Rhode Island [30,32]), and Midwest (e.g., Illinois and Wisconsin [30]) of the United States (see Additional file 2: Table S2).

## Results

### Sequence analyses

Fifty-two different SSCP profiles were detected among the 582 amplicons (Table 2). Amplicons with the same SSCP profile had identical DNA sequences, while those that differed in SSCP profile also differed in DNA sequence by one or more nucleotides. The DNA sequences of the 52 haplotypes varied in length from 404–407 bp and differed from one another by 1–5 bp when aligned over 408 nucleotide positions (Table 2). Genetic variation among haplotypes was detected at 42 (10.3%) positions in the sequence alignment. These mutational differences consisted of 25 transitions, 12 transversions, four indels, and one multiple nucleotide change. There was approximately a 2:1 ratio of purine:pyrimidine transitional changes (18 and seven, respectively).

**Table 2:** **The number of** ***I. scapularis*** **individuals of the different mt 16S rRNA gene haplotypes (HT), and the variable positions in the aligned DNA sequences**

**HT**	**n**	N*	**Alignment position:**																																									
								**1**	**1**	**1**	**1**	**1**	**1**	**1**	**1**	**1**	**1**	**1**	**1**	**1**	**1**	**1**	**1**	**1**	**1**	**2**	**2**	**2**	**2**	**2**	**2**	**2**	**2**	**2**	**2**	**2**	**2**	**2**	**2**	**3**	**3**	**3**	**3**	**3**
			**5**	**6**	**6**	**8**	**9**	**0**	**0**	**0**	**0**	**1**	**5**	**6**	**7**	**7**	**7**	**7**	**7**	**8**	**8**	**8**	**8**	**8**	**9**	**0**	**0**	**0**	**1**	**2**	**3**	**3**	**3**	**3**	**3**	**5**	**6**	**6**	**9**	**0**	**3**	**6**	**6**	**7**
			**7**	**0**	**1**	**8**	**0**	**3**	**4**	**5**	**9**	**2**	**1**	**4**	**3**	**4**	**6**	**7**	**8**	**0**	**1**	**2**	**3**	**4**	**3**	**4**	**5**	**9**	**6**	**2**	**2**	**4**	**6**	**7**	**8**	**8**	**2**	**3**	**6**	**5**	**3**	**4**	**5**	**3**
Is–1	282	45	A	A	A	A	A	-	-	T	T	T	A	G	T	A	A	A	T	T	A	A	G	T	A	G	C	A	G	G	-	G	T	A	T	G	T	T	A	G	C	G	G	G
Is–2	25	11	.	.	.	.	.	-	-	.	.	.	.	.	.	.	.	.	.	.	.	.	A	.	.	.	.	.	.	.	-	.	.	.	.	.	.	.	.	.	.	.	.	.
Is–3	1	1	.	.	.	.	.	-	-	.	.	.	.	.	.	.	.	.	A	.	.	.	A	.	.	.	.	.	.	.	-	.	.	.	.	.	.	.	.	.	.	.	.	.
Is–4	44	18	.	.	.	.	.	-	-	.	.	.	.	.	.	.	T	.	.	.	.	.	.	.	.	.	.	.	.	.	-	.	.	.	.	.	.	.	.	.	.	.	.	.
Is–5	3	3	.	.	.	.	.	-	-	.	.	.	.	.	.	.	T	.	.	.	.	.	.	.	.	.	.	.	.	.	-	.	.	.	.	A	.	.	.	.	.	.	.	.
Is–6	14	10	.	.	.	.	.	-	-	.	.	.	.	.	.	.	.	.	.	.	.	.	.	.	.	.	T	.	.	.	-	.	.	.	.	.	.	.	.	.	.	.	.	.
Is–7	33	9	.	.	.	T	.	-	-	.	.	.	.	.	.	.	.	.	.	.	.	.	A	.	.	.	.	.	.	.	-	.	.	.	.	.	.	.	.	.	.	.	.	.
Is–8	3	2	.	.	.	.	.	-	-	.	.	.	.	.	.	.	.	.	.	.	.	.	.	.	.	.	.	.	.	.	-	A	.	.	.	.	.	.	.	.	.	.	.	.
Is–9	11	8	.	.	.	.	G	-	-	.	.	.	.	.	.	.	.	.	.	.	.	.	.	.	.	.	.	.	.	.	-	.	.	.	.	.	.	.	.	.	.	.	.	.
Is–10	2	2	.	.	.	.	.	-	-	.	.	.	.	.	.	.	.	.	.	.	.	.	.	.	.	.	.	G	.	.	-	.	.	.	.	.	.	.	.	.	.	.	.	.
Is–12	2	2	.	.	.	.	.	-	-	-	.	.	.	.	.	.	.	.	.	A	.	.	.	.	.	.	.	.	.	.	-	.	.	.	.	.	.	.	.	.	.	.	.	.
Is–13	24	17	.	.	.	.	.	-	-	-	.	.	.	.	.	.	.	.	.	.	.	.	.	.	.	.	.	.	.	.	-	.	.	.	.	.	.	.	.	.	.	.	.	.
Is–14	3	3	.	.	.	.	.	-	T	.	.	.	.	.	.	.	T	.	.	.	.	.	.	.	.	.	.	.	.	.	-	.	.	.	.	.	.	.	.	.	.	.	.	.
Is–15	27	11	.	.	.	.	.	-	-	.	.	.	.	.	.	.	.	.	.	.	.	.	.	.	.	.	.	.	.	.	-	.	.	.	.	A	.	.	.	.	.	.	.	.
Is–17	1	1	.	.	.	.	.	-	T	.	.	.	.	.	A	.	.	.	.	.	.	.	.	.	.	.	.	.	.	.	-	.	.	.	.	A	.	.	.	.	.	.	.	.
Is–20	5	5	.	.	.	.	.	-	-	.	.	.	.	.	.	.	.	.	.	.	.	.	.	.	.	.	.	.	.	.	-	.	.	.	.	.	.	.	.	.	.	.	.	.
Is–21	1	1	.	.	.	.	.	-	-	.	.	.	.	.	.	.	.	.	.	.	.	.	.	.	.	.	.	.	.	.	-	A	A	.	.	.	.	.	.	.	.	.	.	.
Is–23	2	2	.	.	.	.	.	-	-	.	.	.	.	.	.	.	.	.	.	.	.	.	.	.	.	.	.	.	.	.	-	.	.	.	C	.	.	.	.	.	.	.	.	.
Is–24	2	2	.	.	.	.	.	-	-	.	.	.	.	.	.	.	.	.	.	.	.	.	.	.	.	A	.	.	.	.	-	.	.	.	.	.	.	.	.	.	.	.	.	.
Is–30	2	2	.	.	.	T	.	-	-	.	.	.	.	.	.	.	T	.	.	.	.	.	.	.	.	.	.	.	.	.	-	.	.	.	.	.	.	.	.	.	.	.	.	.
Is–48	2	2	.	.	.	.	.	-	-	.	.	.	.	.	.	.	.	.	.	.	.	.	A	.	.	.	.	.	.	.	-	.	.	.	.	.	.	.	.	.	.	A	A	.
Is–49	1	1	.	.	.	T	.	-	-	.	.	.	.	.	.	.	.	.	.	.	.	.	A	.	.	.	.	.	.	.	-	.	.	.	.	.	.	.	.	.	.	.	.	A
Is–50	3	3	.	.	.	.	.	-	-	.	.	.	.	.	.	.	T	.	.	.	T	.	.	.	.	.	.	.	.	.	-	.	.	.	.	.	.	.	.	.	.	.	.	.
Is–51	4	3	.	.	.	.	.	-	-	.	.	.	.	.	.	.	.	.	.	.	.	.	.	.	.	.	.	.	.	.	-	.	.	.	.	.	.	C	.	.	.	.	.	.
Is–52	4	4	.	.	.	.	.	-	-	.	.	.	G	.	.	.	.	.	.	.	.	.	.	.	.	.	.	.	.	.	-	.	.	.	.	.	.	.	.	.	.	.	.	.
Is–53	2	2	.	.	.	.	.	-	-	.	.	.	.	.	.	.	.	.	.	.	.	.	.	.	.	.	.	.	.	.	-	.	.	G	.	.	.	.	.	.	.	.	.	.
Is–54	3	3	.	.	.	.	.	-	-	.	.	.	.	.	.	.	.	.	.	.	.	.	.	.	.	.	.	.	.	.	-	.	.	.	.	.	C	.	.	.	.	.	.	.
Is–55	9	5	.	.	.	.	.	-	-	.	C	.	.	.	.	.	.	.	.	.	.	.	.	.	.	.	.	.	.	.	-	.	.	.	.	.	.	.	.	.	.	.	.	.
Is–56	2	2	.	.	G	.	.	-	-	.	.	.	.	.	.	.	.	.	.	.	.	.	.	.	.	.	.	.	.	.	-	.	.	.	.	.	.	.	.	.	.	.	.	.
Is–57	6	4	.	.	.	.	.	-	-	.	.	.	.	.	.	.	.	.	.	.	.	C	.	.	.	.	.	.	.	.	-	.	.	.	.	.	.	.	.	.	.	.	.	.
Is–58	1	1	.	.	.	.	.	-	-	.	.	.	.	.	.	.	.	.	.	.	.	.	.	.	.	.	.	.	.	.	-	.	.	.	.	.	.	.	.	A	.	.	.	.
Is–59	2	1	.	.	.	.	.	-	-	.	.	.	.	.	.	.	.	.	.	.	.	.	.	.	.	.	.	.	.	.	-	.	.	.	.	.	.	.	T	.	.	.	.	.
Is–60	2	1	.	.	.	.	.	-	-	.	.	.	.	.	.	.	.	T	.	.	.	.	.	.	.	.	.	.	.	.	-	.	.	.	.	.	.	.	.	.	.	.	.	.
Is–61	1	1	.	.	.	T	.	-	-	.	.	.	.	.	.	.	.	.	.	.	.	.	.	.	.	.	.	.	.	.	-	.	.	.	.	.	.	.	.	.	.	.	.	.
Is–62	4	4	G	.	.	.	.	-	-	.	.	.	.	.	.	.	.	.	.	.	.	.	.	.	.	.	.	.	.	.	-	.	.	.	.	.	.	.	.	.	.	.	.	.
Is–63	20	12	.	.	.	.	.	-	T	.	.	.	.	.	.	.	.	.	.	.	.	.	.	.	.	.	.	.	.	.	-	.	.	.	.	.	.	.	.	.	.	.	.	.
Is–64	1	1	.	.	.	.	.	-	-	.	.	.	.	.	.	.	.	.	.	.	.	.	.	.	.	.	.	.	.	.	-	.	A	.	.	.	.	.	.	.	.	.	.	.
Is–65	1	1	.	.	.	.	.	-	-	.	.	.	.	.	.	.	.	.	.	.	.	.	.	.	.	.	.	.	.	A	-	.	.	.	.	.	.	.	.	.	.	.	.	.
Is–66	1	1	.	.	.	.	.	-	-	.	.	.	.	A	.	.	.	.	.	.	.	.	.	.	.	.	.	.	.	.	-	.	.	.	.	.	.	.	.	.	.	.	.	.
Is–67	1	1	.	.	.	.	.	-	-	.	.	C	.	.	.	.	.	.	.	.	.	.	.	.	.	.	.	.	.	.	-	.	.	.	.	.	.	.	.	.	.	.	.	.
Is–68	1	1	.	.	.	.	.	-	-	.	.	.	.	.	.	.	.	.	.	.	.	.	.	.	.	.	.	.	.	.	-	.	.	.	.	A	.	.	.	.	T	.	.	.
Is–69	3	1	.	.	.	.	.	-	-	.	.	.	.	.	.	.	.	.	.	.	.	.	.	.	.	.	.	.	.	.	T	.	.	.	.	.	.	.	.	.	.	.	.	.
Is–70	2	2	.	.	.	.	.	-	-	.	.	.	.	.	.	.	T	.	.	.	.	.	.	.	.	.	.	.	.	.	-	.	.	.	.	.	.	.	G	.	.	.	.	.
Is–71	1	1	.	.	.	.	.	-	-	.	.	.	.	.	.	.	.	.	.	.	.	.	.	.	G	.	.	.	.	.	-	.	.	.	.	.	.	.	.	.	.	.	.	.
Is–72	2	2	.	.	.	.	.	-	-	.	.	.	.	.	.	G	.	.	.	.	.	.	.	.	.	.	.	.	.	.	-	.	.	.	.	.	.	.	.	.	.	.	.	.
Is–73	2	2	.	.	.	.	.	-	-	-	.	.	.	.	.	.	T	.	.	.	.	.	.	.	.	.	.	.	.	.	-	.	.	.	.	.	.	.	.	.	.	.	.	.
Is–74	6	6	.	.	.	.	.	-	-	.	.	.	.	.	.	.	.	.	.	.	.	.	A	A	.	.	.	.	.	.	-	.	.	.	.	.	.	.	.	.	.	.	.	.
Is–75	1	1	.	.	.	.	.	T	T	.	.	.	.	.	.	.	.	.	.	.	.	.	.	.	.	.	.	.	.	.	-	.	.	.	.	.	.	.	.	.	.	.	.	.
Is–76	3	3	.	T	.	.	.	-	-	.	.	.	.	.	.	.	.	.	.	.	.	.	.	.	.	.	.	.	.	.	-	.	.	.	.	.	.	.	.	.	.	.	.	.
Is–77	2	2	.	.	.	T	.	-	-	-	.	.	.	.	.	.	.	.	.	.	.	.	A	.	.	.	.	.	.	.	-	.	.	.	.	.	.	.	.	.	.	.	.	.
Is–78	1	1	.	.	.	.	.	-	-	.	.	.	.	.	.	.	.	.	.	.	.	.	.	.	.	.	.	.	C	.	-	.	.	.	.	.	.	.	.	.	.	.	.	.
Is–79	1	1	.	.	.	T	.	-	T	.	.	.	.	.	.	.	.	.	.	.	.	.	A	.	.	.	.	.	.	.	-	.	.	.	.	.	.	.	.	.	.	.	.	.

A dot (.) at an alignment position indicates the same nucleotide as in the sequence of haplotype Is-1, while a dash (−) represents a deletion.

Abbreviation: *n* = number of individuals with same SSCP profile, N* = number of amplicons sequenced.

### Sequence variation in relation to the secondary structure

**Figure 2:**
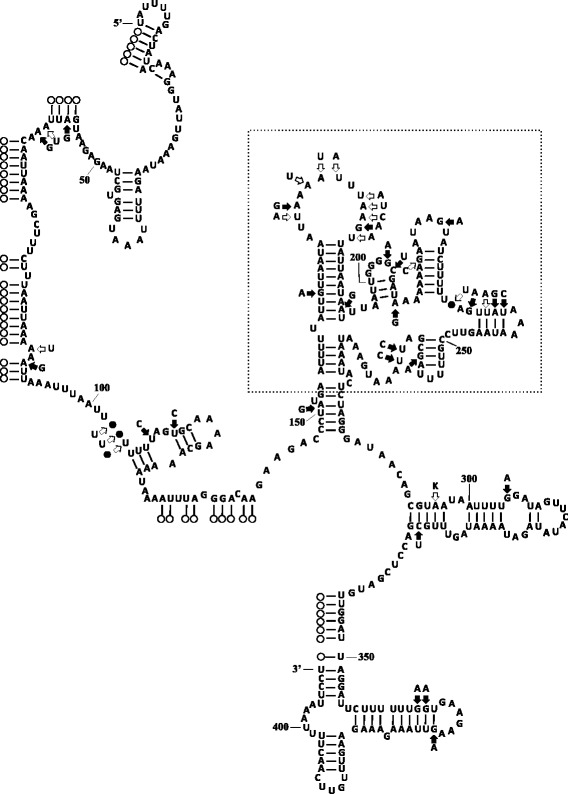
**The predicted secondary structure of Domains IV and V of the mt 16S rRNA gene for haplotype Is-1 of** ***I. scapularis*** **.** Open circles indicate putative nucleotides within other domains of the gene [38]. Closed circles indicate deletions in the sequence of haplotype Is-1 compared to the DNA sequences some other haplotypes of *I. scapularis* (see Table 2). Solid arrows indicate purine and pyrimidine transitional changes, while open arrows indicate transversional mutations, multiple changes, or indels in the DNA sequences of the other 16S haplotypes detected in this study relative to the DNA sequence of haplotype Is-1. The box indicates the hypervariable region as defined by Smith and Bond [53].

Twenty-six (61.9%) of the 42 mutational changes occurred at unpaired sites (e.g., end loops and internal loops) in the predicted secondary structure of the mt 16S rRNA gene, while another nine mutational changes represented partial-compensatory base-pair changes that maintained the secondary structure (Figure 2). A majority of the mutational changes occurred within the hypervariable region (alignment positions 154 to 279; see Figure 2). This region comprised 126 (30.9%) of the nucleotides in the 3’ terminal end of the mt 16S rRNA gene, but contained 25 (59.5%) of the variable nucleotide positions among haplotypes. Thus, the proportion of variable positions in the hypervariable region was 19.8%. In contrast, there was a significantly (χ^2^_1_ = 17.99, *P* <0.001) lower proportion of variable positions (6.0%; 17 of 282 positions) in the 5’ and 3’ regions flanking the hypervariable region, which represented 69.1% of the 408 total nucleotides at the 3’ terminal end of the gene. These two flanking regions contained 17 (40.5%) of the 42 variable nucleotide positions among sequence types.

### Phylogenetic analysis

The tree produced from the NJ analysis of the sequence data (Additional file 3: Figure S1) separated the 16S haplotypes of *I. scapularis* into two major clades; the Southern clade and American clade. This was supported by the results of the bootstrap analysis. However, there was little statistical support for the different groups within the American clade. All 582 *I. scapularis* characterized in the present study belonged to the American clade.

### Population genetic analyses

**Figure 3:**
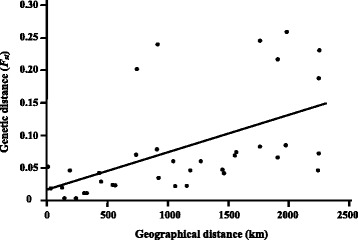
**Pair-wise comparison of the genetic (** ***F***_***ST***_ **) and geographical (km) distances among the nine established populations of** ***I. scapularis*** **in Canada and the United States.**

The haplotype diversities and nucleotide diversities of *I. scapularis* within the nine established populations ranged from 0.5994 to 0.7856, and 0.00191 to 0.00357, respectively (Table 3). For six populations, the 7–14 haplotypes detected within each population was not significantly different from the expected number based on Chakraborty’s test, whereas significantly more haplotypes were detected than expected in all three populations from Minnesota (Table 3). The results of the Tajima’s test for neutrality indicated that there were significant negative departures from zero for the three tick populations in Minnesota and the population in Pembina Valley Provincial Park, Manitoba (Table 3). Similarly, the *F*_*S*_ statistics of Fu revealed that the *F*_*S*_ values for five populations (three in Minnesota and two in Manitoba) differed significantly from zero. A single haplotype (Is-1) was found in all nine *I. scapularis* populations and comprised 34-63% of the ticks within each population (Figure 1). The second most common haplotype differed among populations and represented between 7-32% of the ticks within each population. Comparison of the *F*_*ST*_ values revealed significant differences between most pairs of populations except between some populations in Manitoba and Minnesota (Table 4). The results of the AMOVA test (Table 5) also indicated strong genetic structure among populations both within and among different geographical regions. Most of the variance (94.3%) occurred within populations. Nonetheless, there were many shared haplotypes among populations in different geographical regions (Figure 1). The results of the Mantel Test (Figure 3), which compared pair-wise *F*_*ST*_ values as a factor of geographical distance between tick populations, showed that there was a significant association between genetic and geographical distances (*b* = 0.000058, *r*^*2*^ = 0.299, *P* = 0.002).

**Table 3:** **Haplotype diversity and nucleotide diversity estimates, and neutrality test results of nine established populations of** ***I. scapularis***

**Population**	**N**	**S**	***h***	***π***	**Neutrality tests**		**Chakraborty’s test**	
					**Tajima’s**	**Fu’s**	**No of haplotypes:**	
					**D**	***Fs***	**Exp.**	**Obs.**
Pembina Valley Provincial Park, MB	46	12	0.7807	0.00292	−1.7018^*^	−7.3735^***^	8.5	12
Stanley Trail, MB	44	7	0.6945	0.00217	−1.0354	−3.7782^*^	6.2	8
Itasca State Park, MN	56	10	0.5994	0.00191	−1.7473^*^	−8.2716^***^	5.0	11^**^
Camp Ripley, MN	56	15	0.6461	0.00240	−2.0751^**^	−11.5757^***^	5.7	14^***^
St. Croix State Park, MN	56	13	0.6656	0.00213	−1.9112^**^	−12.7498^***^	6.0	14^***^
Point Pelee National Park, ON	46	6	0.6019	0.00206	−1.2915	−2.7386	4.8	7
Long Point Provincial Park, ON	108	13	0.7856	0.00318	−1.2840	−5.5321	11.0	13
Trustom Pond, RI	50	7	0.7273	0.00316	−0.0610	−1.1404	7.2	7
Hazard Island, RI	50	8	0.7731	0.00357	−0.0852	−3.4150	8.5	10

Abbreviations: N = sample size, S = no. of polymorphic sites, *h* = haplotype diversity and *π* = nucleotide diversity. Significance levels: *P <0.05, **P <0.01 and ***P <0.001. For the state and province abbreviations, see Table 1.

**Table 4:** **Pair-wise comparisons of geographical (km; upper diagonal) and genetic (** ***F***_***ST***_ **values; lower diagonal) distances among established populations of** ***I. scapularis***

**Population**	**1**	**2**	**3**	**4**	**5**	**6**	**7**	**8**	**9**
1 Pembina Valley Provincial Park, MB	-	29	307	426	543	1452	1554	2241	2246
2 Stanley Trail, MB	0.0187	-	326 447	560	1465	1565	2247	2252	
3 Itasca State Park, MN	0.0114	0.0110	-	123	238	1157	1272	1978	1983
4 Camp Ripley, MN	0.0418^***^	0.0291^***^	0.0214^*^	-	145	1061	1186	1904	1909
5 St. Croix State Park, MN	0.0240^*^	0.0231^*^	0.0032	0.0033	-	921	1042	1758	1763
6 Point Pelee National Park, ON	0.0474^**^	0.0416^***^	0.0219^*^	0.0225^**^	0.0343^***^	-	188	909	914
7 Long Point Provincial Park, ON	0.0692^***^	0.0743^***^	0.0604^***^	0.0459^***^	0.0604^***^	0.0462^***^	-	738	743
8 Trustom Pond, RI	0.0461^***^	0.0720^***^	0.0847^***^	0.0664^***^	0.0830^***^	0.0788^***^	0.0701^***^ -	5	
9 Hazard Island, RI	0.1873^***^	0.2305^***^	0.2590^***^	0.2160^***^	0.2451^***^	0.2393^***^	0.2012^***^	0.0516^***^	-

Significance levels: *P <0.05, **P <0.01 and ***P <0.001. For the state and province abbreviations, see Table 1.

**Table 5:** **Analysis of Molecular Variance (AMOVA) for nine established populations of** ***I. scapularis*** **from Canada and the United States**

**Variance component**	***df***	**% variance**	**Fixation index**	***P***
Among regions^a^	3	3.8	Φ_CT_ =0.03791	< 0.0001
Among populations within regions	5	1.9	Φ_SC_ =0.01947	< 0.005
Within populations	503	94.3	Φ_ST_ =0.05665	< 0.0001

^a^Regions = Manitoba (PVPP & ST), Ontario (PPNP & LPPP), Minnesota (ISP, CSP & CP) and Rhode Island (TP & HI).

**Figure 4:**
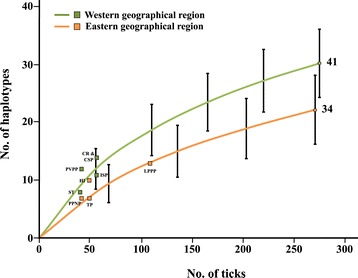
**Rarefaction curves with 95% confidence intervals of haplotype diversity for populations of** ***I. scapularis*** **in the western and eastern geographical regions.** The numbers next to the curves indicate the total estimated number of haplotypes using the non-parametric Chao 2 estimator. See list of abbreviations for the complete names of localities of the tick populations.

A total of 30 haplotypes were detected in five populations in the western geographical region, while 22 haplotypes were detected in the four populations in the eastern geographical region. The Chao 2 estimates of haplotype richness were higher for the western populations than in the eastern populations (41 and 34 haplotypes, respectively; Figure 4). However, the results of the rarefaction analyses showed that, although the curves for both the western and eastern populations of *I. scapularis* did not converge on an asymptote, there was no significant difference in haplotype diversity between populations of the two geographical areas, as there was overlap in the 95% confidence intervals of the two curves (Figure 4). Similarly, when the data for all populations were pooled, the rarefaction curve (see Additional file 4: Figure S2) did not reach an asymptote or approach the Chao 2 estimate of the haplotype diversity (i.e., 82 haplotypes). Therefore, only 45 (55%) of the expected total number of haplotypes were detected in the nine established populations of *I. scapularis*.

### Geographical variation

**Figure 5:**
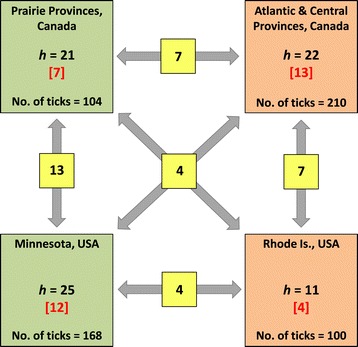
**The number of mt 16S rRNA gene haplotypes (** ***h*** **) of** ***I. scapularis*** **found in different geographical regions of Canada, and the Midwest and Northeast of the United States.** The numbers inside parentheses indicate the number of haplotypes only found exclusively in a geographical region. Also shown is the number of haplotypes (inside yellow boxes) shared among geographical regions.

Fifteen (29%) of the 52 haplotypes detected among the adventitious ticks and individuals from established populations in the present study were each represented by a single tick, while four haplotypes were detected in western and eastern Canada, and in the Midwest and Northeast of the United States (Figure 5 and Additional file 5: Table S3). Thirty-two haplotypes were detected among ticks from the United States; however, only four (13%) were present in populations in the Midwest and Northeast. Ticks collected from western Canada had the highest similarity, based on the proportion of shared haplotypes, with the ticks from the Midwest of the United States (13 of 33; 39%) rather than those in eastern Canada (7 of 36; 19%) or the Northeast of the United States (5 of 27; 19%). The proportion of shared haplotypes between ticks from eastern Canada and the Northeast of the United States (7 of 26; 27%) was greater than that between ticks from the Midwest of the United States (6 of 41; 15%) or western Canada (19%).

**Figure 6:**
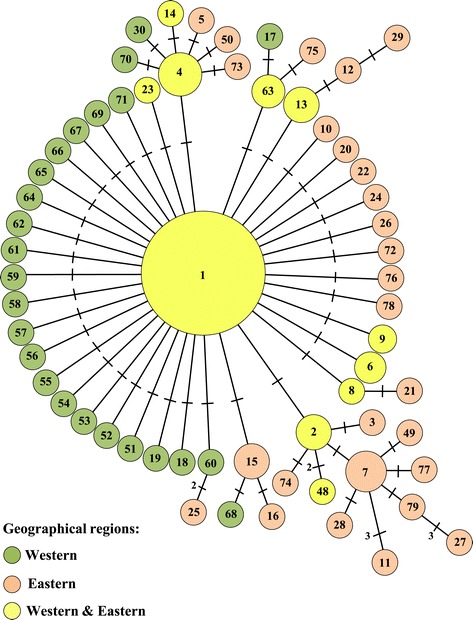
**A minimum spanning network tree depicting the relationships of the different mt 16S rRNA gene haplotypes of** ***I. scapularis*** **in the American clade detected in this and other studies [** 30 **-** 32,34 **].** Crossbars between two haplotypes indicate one nucleotide difference in DNA sequences unless stated otherwise. The size of each circle is proportional to the number of ticks of that haplotype (for this study only).

The minimum spanning network tree depicting the relationships among haplotypes of the American clade is shown in Figure 6. Most (82%) haplotypes were only found in either the western or eastern geographical region (27 and 24 haplotypes, respectively). Haplotype Is-1, the most common haplotype in both geographical regions (see Additional file 5: Table S3), represented the central haplotype of the star-shaped network tree. All other haplotypes differed from the central haplotype by 1–6 bp. Thirty-seven haplotypes differed in sequence from the central haplotype by a single nucleotide mutation. These included five of the seven most common haplotypes detected in the present study (Is-2, Is-4, Is-13, Is-15, and Is-63; Additional file 5: Table S3). Each of these haplotypes represented a link (secondary node) from the central haplotype to other haplotypes (Figure 6). Six of the eight most common haplotypes were detected in *I. scapularis* populations in both the western and eastern geographical regions. Another common haplotype present in eastern populations (Is-7) represented a tertiary node in the haplotype network to six other haplotypes that were also only detected in eastern tick populations.

## Discussion

Fifty-two haplotypes of the mt 16S rRNA gene were detected among the 582 *I. scapularis* individuals collected from southern Canada, and the Midwest (Minnesota) and Northeast (Rhode Island) of the United States. All 52 haplotypes belonged to the American clade, as defined by Qiu *et al*. [32]. The lack of *I. scapularis* individuals of the Southern clade in northern United States and southern Canada is consistent with the findings of other studies [30-32,34]. The total number of haplotypes detected in the present study was greater than the 7–29 haplotypes detected in other studies of the American clade [18,30-34]), and included 30 haplotypes not reported previously. The difference in number of the haplotypes detected among studies may be a consequence of differences in sample sizes. For example, the number of haplotypes among *I. scapularis* individuals collected from Camp Riley in Morrison County, Minnesota (14 haplotypes), Trustom Pond and Hazard Island in Washington County, Rhode Island (11 haplotypes), and Long Point Provincial Park, Ontario (13 haplotypes) were two to three times greater than that reported previously in these areas (3, 4 and 6 haplotypes, respectively) [31,32,34]; however, at least twice as many ticks were sampled from each of these localities in the present study. Nonetheless, the results of the present study indicate that genetic diversity in *I. scapularis* from northern United States and southern Canada, based on DNA sequences of the mt 16S rRNA gene, is considerably greater than previously thought. Furthermore, there may be a large number of undetected haplotypes within the sampled areas because the Chao 2 estimate of the total number of expected haplotypes (i.e., 82) was greater than the 52 haplotypes detected. This is likely given that 15 (29%) of the haplotypes can be considered as rare because only one tick of each of these haplotypes was detected in the present study.

Nucleotide diversities within established populations of *I. scapularis* were low (0.002-0.004), while haplotype diversities were relatively high (0.60-0.79) compared to those of some other species of *Ixodes* in North America [48-51]. For example, only 1–4 haplotypes of the mt 16S rRNA gene have been detected within populations of *Ixodes angustus*, *Ixodes kingi* and *Ixodes sculptus* [48-51] compared to the 7–14 haplotypes among individuals in populations of *I. scapularis*. Biological differences among these tick species are likely explanations for the differences in haplotype number. For instance, *I. angustus*, *I. kingi* and *I. sculptus* parasitize primarily small mammals (e.g., mice, shrews, voles, ground squirrels and/or pocket gophers [49-52], which provide limited dispersal distances for ticks. In contrast, *I. scapularis* parasitizes a wider diversity of animals including passerine birds [20,52], hosts that are known to carry *I. scapularis* larvae and nymphs over large distances [7,29]. Therefore, increased dispersal distance provides a greater opportunity for transfer of ticks representing different maternal lineages (haplotypes) from one population to another.

Variation in the DNA sequences among *I. scapularis* individuals was also examined in relation to the predicted secondary structure of Domains IV and V of the mt 16S rRNA gene. Over half (57%) of the nucleotide variation occurred within the “hypervariable” region of Domain V. Mutational alterations in DNA sequence, both within and among species of arthropod, have been shown previously to be more frequent within this part of the gene than in the flanking regions [48,53-58]. This suggests that there are fewer structural constraints for mutational changes within the hypervariable region than in other parts of Domain V [54]. DNA sequence variation in *I. scapularis* at unpaired sites (62%) was also greater than that at sites involved in base pairing (38%) in the secondary structure. This difference may also be associated with reduced structural constraints for mutational alterations at nucleotide positions not involved in base pairing. In addition, substitutions at nucleotide positions involved in base pairing were more often partial compensatory base changes, rather than non-compensatory changes, hence maintaining the integrity of the secondary structure of the mt 16S rRNA gene. This is in agreement with the general patterns of mutational change in rRNA genes [59].

The number of nucleotide differences in DNA sequence among the 52 haplotypes ranged from 1–5 bp; however, this difference increased to 9 bp when an additional 10 haplotypes of the American clade [30-32,34] were added to the analyses. Seven haplotypes (Is-1, Is-2, Is-4, Is-7, Is-13, Is-15 and Is-63) comprised 78% of the ticks collected in the present study. Of these, haplotype Is-1 was the most common (49%), which was consistent with the findings of other studies on *I. scapularis* conducted in southern Canada [34], and the Midwest and Northeast of the United States [30,32]. Haplotype Is-1 also represented the central haplotype of the minimum spanning network. Some of the other common haplotypes represented secondary or tertiary nodes in the network. This suggests that most haplotypes were derived from the central haplotype or one of the secondary haplotypes, as a consequence of a single mutational change in the DNA sequence of the mt 16S rRNA gene. Furthermore, the star-shaped pattern of the network tree for *I. scapularis* is indicative of rapidly expanding populations [18,32], which is in agreement with the relatively recent establishment of populations of *I. scapularis* in the Midwest of the United States [4,60] and southern Canada [7,9,28]. Furthermore, for the populations in Manitoba and Minnesota, there were significant negative departures from zero for both the Tajima’s D and Fu’s F_s_ tests suggesting population expansion at these localities.

Range expansion and the establishment of geographically isolated populations of *I. scapularis* into southern Canada have been attributed to the transportation of large numbers of larvae and nymphs from the United States by migratory passerine birds [7,29]. However, the geographical origins of these populations are unknown. Krakowetz *et al*. [34] reported differences in the genetic structure of *I. scapularis* between a population in southeastern Manitoba and several populations in southern Ontario and Nova Scotia. Subsequently, Mechai et al. [61] reported differences in the frequency of haplotypes of the mt cytochrome C oxidase subunit 1 gene (*cox1*) among blacklegged ticks from different geographical regions in Canada. It was proposed that *I. scapularis* populations in different geographical regions of southern Canada may be derived from populations in different regions of the United States and associated with the different routes (flyways) taken by passerines during their spring migration [34]. Scott *et al*. [29] also suggested that there was an association between the presence of *I. scapularis* and other introduced tick species in different regions of Canada and the different flyways of migratory passerines. Thus, blacklegged ticks introduced into the Prairie Provinces of Canada (Manitoba, Saskatchewan and Alberta) may have originated from endemic populations in the Midwest of the United States (Minnesota and Wisconsin), while those introduced into the Central Provinces of Canada (Ontario and Quebec) may have originated from endemic populations primarily in the Northeast of the United States (Connecticut, Pennsylvania, New York, Massachusetts, Rhode Island, Maine and New Hampshire), but also from resident populations in parts of the Midwest [29,34]. Northeastern United States was also suggested as the most likely origin of blacklegged ticks introduced into the Atlantic Provinces (Nova Scotia, New Brunswick, Newfoundland and Prince Edward Island) [29,34]. The latter is supported by the results of the present study. For example, 38% of ticks from an established population in Lunenburg (Nova Scotia) were haplotype Is-12, a haplotype that had not been previously reported from other regions of Canada or in the United States [34]; however, this haplotype was detected in Rhode Island (present study). Furthermore, nine (75%) of the 12 haplotypes found in the Atlantic Provinces were also detected in Rhode Island, seven of which have also been found in other parts of the Northeast of the United States [32].

The results of the AMOVA test also revealed statistically significant genetic structuring of *I. scapularis* populations both within and among different geographical regions; however, the presence of several shared haplotypes among populations supports the hypothesis of gene flow among populations. Nonetheless, 26 (79%) of the 33 haplotypes found in the western region (Prairie Provinces and Minnesota) were not found in the eastern region (Central and Atlantic Provinces, and Rhode Island), while 19 of the 26 (73%) haplotypes in the east were not found in Minnesota or the Prairie Provinces. In addition, only 8 (32%) of the 25 haplotypes found in the three populations in Minnesota have been reported previously from the Northeast of the United States [30,32], which includes four haplotypes present in Rhode Island. There was also a significant positive correlation between the geographical (km) distances among populations and the magnitude of genetic differences (*F*_*ST*_ values). Statistical analyses of the *F*_*ST*_ data also showed significant differences in the genetic structure between some populations of *I. scapularis*. For example, there was a significant difference in the population genetic structure of *I. scapularis* from Hazard Island and Trustom Pond, two localities in South Kingstown (Rhode Island) separated by a distance of only 5 km. The reason why the genetic structure of these two tick populations differs is unclear. Although fewer haplotypes were detected at Trustom Pond, six of the seven haplotypes in this population were also detected in the population at Hazard Island. In addition, the results of the Chakraborty’s test revealed no significant difference in the number of observed and expected haplotypes for either population. In contrast, there were no significant differences between the populations in Itasca State Park (Minnesota), Pembina Valley Provincial Park and Stanley Trail (Manitoba). There were also no differences between the populations in Itasca State Park and St. Croix State Park, or between those in Camp Ripley and St. Croix State Park (Minnesota). A comparison of the haplotypes among adventitious ticks and those in established populations found in the Prairie Provinces with those in other geographical areas (Figure 6) revealed a greater similarity to *I. scapularis* in Minnesota than to those in the Central and Atlantic Provinces of Canada or Rhode Island based on the proportion of shared haplotypes. These results provide some support for the hypothesis that the *I. scapularis* populations in southern Manitoba are derived from established populations in the Midwest of the United States. However, a large proportion (58%) of the *I. scapularis* individuals collected in all three regions of southern Canada (Prairie, Central and Atlantic Provinces) were of a haplotype (Is-1, Is-2, Is-4 and Is-6) that also occurs in both the Midwest and Northeast of the United States [30,32]. Therefore, other genetic markers (e.g., 12S rRNA gene [31] or *cox1*; [61]), in addition to the mt 16S rRNA gene, are needed to determine the geographical origin of *I. scapularis* introductions into southern Canada.

## Conclusion

In conclusion, genetic variation within *I. scapularis* was greater than previously demonstrated based on the DNA sequence analyses of the mt 16S rRNA gene. Furthermore, a large number of rare haplotypes may still remain undetected. The results also indicated significant differences in genetic diversity both within and among populations from different geographical regions. There was also a significant positive relationship between the genetic differences between populations and the geographical distances that separated them. There was some evidence to support the hypothesis that *I. scapularis* in the Prairie Provinces of Canada are derived from individuals introduced from the Midwest of the United States, while those in the Atlantic and Central Provinces are derived from individuals that originated in the Northeast of the United States. However, the geographical origins of a large proportion of *I. scapularis* found in the different areas of southern Canada could not be inferred because they were of a haplotype that occurs in both the Midwest and Northeast of the United States. Therefore, additional studies are needed to explore other genetic markers that may be useful for understanding the trajectories of spread of *I. scapularis* and its pathogens on a finer scale.

## Additional files

Additional file 1: Table S1. The number of nymphal or adult male and female *I. scapularis* collected in different years from 11 geographical regions in North America. Additional file 2: Table S2. Comparison of the haplotype designations of *I. scapularis* for the mt 16S rRNA gene used in the minimum spanning network tree (see Figure 6) of the present study in relation to those (e.g., haplotype, specimen number or GenBank accession no.) used in previous studies. Additional file 3: Figure S1. Neighbor-joining tree depicting the relationships of the 52 mt 16S rDNA haplotypes of *I. scapularis* detected in the present study. Also included are an additional 10 haplotypes from other studies of the American [32,34] and Southern clades [31,32]. Numbers above branches indicate the bootstrap values (>70%). The scale bar represents the inferred substitutions per nucleotide site. Haplotypes identical to those of haplotypes A-M of Qiu *et al*. [32] are indicated by an *. Additional file 4: Figure S2. Rarefaction curve (with 95% confidence intervals) of the haplotype diversity for the *I. scapularis* populations sampled in the present study. The number next to the curve indicates the total estimated number of haplotypes using the non-parametric Chao 2 estimator. See list of abbreviations for the complete names of localities of the tick populations. Additional file 5: Table S3. The number of *I. scapularis* of the different mt 16S rRNA gene haplotypes collected from different geographical regions.

## Abbreviations

CR: Camp Ripley
CSP: St. Croix State P
*h*: Haplotype diversity
ISP: Itasca State Park
LPPP: Long Point Provincial Park
LSU: Large subunit
mt: Mitochondrial
NJ: Neighbor-joining
PCR: Polymerase chain reaction
π: Nucleotide diversity
PPNP: Point Pelee National Park
PVPP: Pembina Valley Provincial Park
rRNA: Ribosomal RNA
HI: Hazard Island
SSCP: Single-strand conformation polymorphism
SSU: Small subunit
ST: Stanley Trail
TP: Trustom Pond
