# Supplementary material for: Genetic variation in the mitochondrial 16S ribosomal RNA gene of Ixodes scapularis (Acari: Ixodidae)
Source: Parasit Vectors. 2014 Nov 28;7:530. doi: 10.1186/s13071-014-0530-6 (PMC4258262; doi:10.1186/s13071-014-0530-6)
Supplement: Additional file 1: Table S1. — The number of nymphal or adult male and female I. scapularis collected in different years from 11 geographical regions in North America. [file 13071_2014_530_MOESM1_ESM.docx]

**Table S1.** **The number of nymphal or adult male and female *I. scapularis* collected in different years from 11 geographical regions in North America**

_________________________________________________________________________________________________

Region Collection year(s) No. of No. of No. of Total

nymphs females males

_________________________________________________________________________________________________

Canada

Prairie

Alberta (AB) 2011 0 2 0 2

Saskatchewan (SK) 2009, 2010 0 6 0 6

Manitoba (MB) 2010, 2011 0 46 50 96

Central

Ontario (ON) 2000, 2006-2009, 2011 0 102 64 166

Quebec (QC) 2005-2007, 2009-2011 0 21 1 22

Atlantic

New Brunswick (NB) 2007, 2009-2011 0 6 0 6

Prince Edward Island (PE) 2006, 2007, 2011 0 3 0 3

Nova Scotia (NS) 2006-2011 0 11 0 11

Newfoundland (NL) 2011 0 2 0 2

U.S.A.

Midwest

Minnesota (MN) 2008 0 84 84 168

Northeast

Rhode Island (RI) 2009, 2010 100 0 0 100

Total 100 283 199 582

_________________________________________________________________________________________________
