# Supplementary material for: Genetic variation in the mitochondrial 16S ribosomal RNA gene of Ixodes scapularis (Acari: Ixodidae)
Source: Parasit Vectors. 2014 Nov 28;7:530. doi: 10.1186/s13071-014-0530-6 (PMC4258262; doi:10.1186/s13071-014-0530-6)
Supplement: Additional file 2: Table S2. — Comparison of the haplotype designations of I. scapularis for the mt 16S rRNA gene used in the minimum spanning network tree (see Figure 6) of the present study in relation to those (e.g., haplotype, specimen number or GenBank accession no.) used in previous studies. [file 13071_2014_530_MOESM2_ESM.docx]

**Table S2**  **Comparison of the haplotype designations of *I. scapularis* for the mt 16S rRNA gene used in the minimum spanning network tree (see Figure 7) of the present study in relation to those (e.g., haplotype, specimen number or GenBank accession no.) used in previous studies**

_____________________________________________________________________________

Haplotype^a^ Qiu et al. [31]^b^ Norris et al. [30] Rich et al. [29]^c^

_____________________________________________________________________________

Is-1 Hap F L43862, L43875 U26605 (IL94)

Is-2 Hap A U26612 (ME92b)

Is-3 Hap C

Is-4 Hap D L43857 U26613 (MS93)

Is-5 Hap E

Is-6 Hap G

Is-7 Hap H U26600 (CT93), U26608 (MA92c)

Is-8 U26617 (NC94)

Is-9 U26611 (ME92a)

Is-10 L43867

Is-11 L43873

Is-20 Hap B

Is-21 Hap I

Is-22 Hap J

Is-23 Hap K

Is-24 Hap L

Is-25 NC1_6

Is-26 NC2_22

Is-27 NC2_29

Is-28 NY2_11 U26600 (CT93), U26608 (MA92c)

Is-29 MA1

Is-30 L43858

Is-48 U26612 (ME92b)

Is-49 U26600 (CT93), U26608 (MA92c)

Is-80 Hap M L43855, L43856

Is-81 Hap N

Is-82 Hap O

Is-83 L43854

Is-84 L43861

Is-85 L43863

Is-86 L43865

Is-87 L43866

_____________________________________________________________________________

^a^ Haplotypes Is-12 to Is-19 and Is-50 to Is-79 not detected in the other studies.

^b^ Accession numbers: AF309011–AF309030

^c^ Based on sequences of only ≈342 bp (i.e., missing 18 bp at 5’ end and 51 bp at 3’ end).
