# Supplementary material for: Genetic variation in the mitochondrial 16S ribosomal RNA gene of Ixodes scapularis (Acari: Ixodidae)
Source: Parasit Vectors. 2014 Nov 28;7:530. doi: 10.1186/s13071-014-0530-6 (PMC4258262; doi:10.1186/s13071-014-0530-6)
Supplement: Additional file 3: Figure S1. — Neighbor-joining tree depicting the relationships of the 52 mt 16S rDNA haplotypes of I. scapularis detected in the present study. Also included are an additional 10 haplotypes from other studies of the American [32,34] and Southern clades [31,32]. Numbers above branches indicate the bootstrap values (>70%). The scale bar represents the inferred substitutions per nucleotide site. Haplotypes identical to those of haplotypes A-M of Qiu et al. [32] are indicated by an *. [file 13071_2014_530_MOESM3_ESM.pptx]

## Slide 1
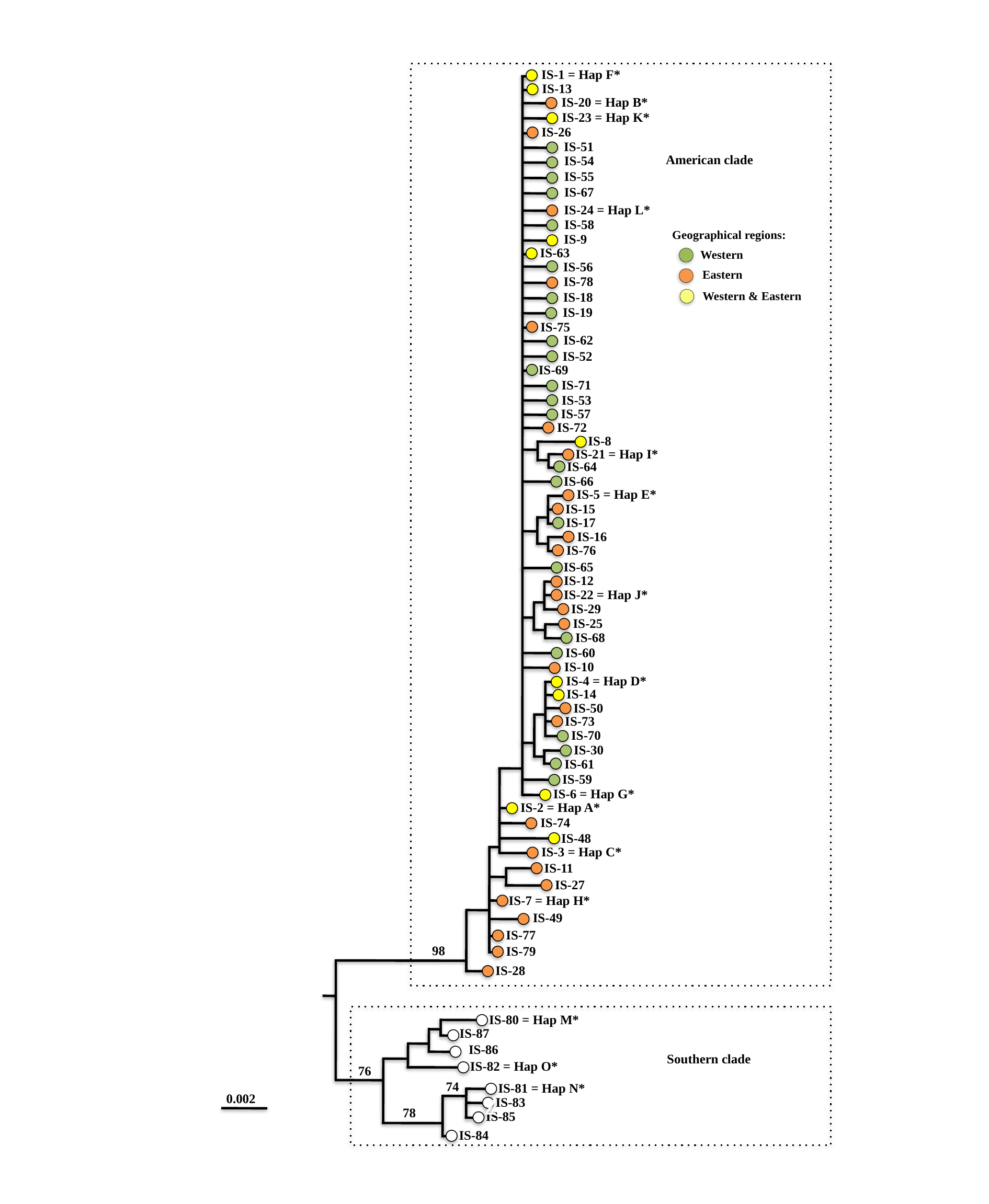

IS-1 = Hap F*
IS-13
IS-20 = Hap B*
IS-23 = Hap K*
IS-26
IS-51
American clade
IS-54
IS-55
IS-67
IS-24 = Hap L*
IS-58
Geographical regions:
IS-9
IS-63
 Western
IS-56
 Eastern
IS-78
 Western & Eastern
IS-18
IS-19
IS-75
IS-62
IS-52
IS-69
IS-71
IS-53
IS-57
IS-72
IS-8
IS-21 = Hap I*
IS-64
IS-66
IS-5 = Hap E*
IS-15
IS-17
IS-16
IS-76
IS-65
IS-12
IS-22 = Hap J*
IS-29
IS-25
IS-68
IS-60
IS-10
IS-4 = Hap D*
IS-14
IS-50
IS-73
IS-70
IS-30
IS-61
IS-59
IS-6 = Hap G*
IS-2 = Hap A*
IS-74
IS-48
IS-3 = Hap C*
IS-11
IS-27
IS-7 = Hap H*
IS-49
IS-77
98
IS-79
IS-28
IS-80 = Hap M*
IS-87
IS-86
Southern clade
IS-82 = Hap O*
76
74
IS-81 = Hap N*
0.002
IS-83
z
78
IS-85
IS-84
