# Supplementary figures and images for: Genetic variation in the mitochondrial 16S ribosomal RNA gene of Ixodes scapularis (Acari: Ixodidae)
Source: Parasit Vectors. 2014 Nov 28;7:530. doi: 10.1186/s13071-014-0530-6 (PMC4258262; doi:10.1186/s13071-014-0530-6)

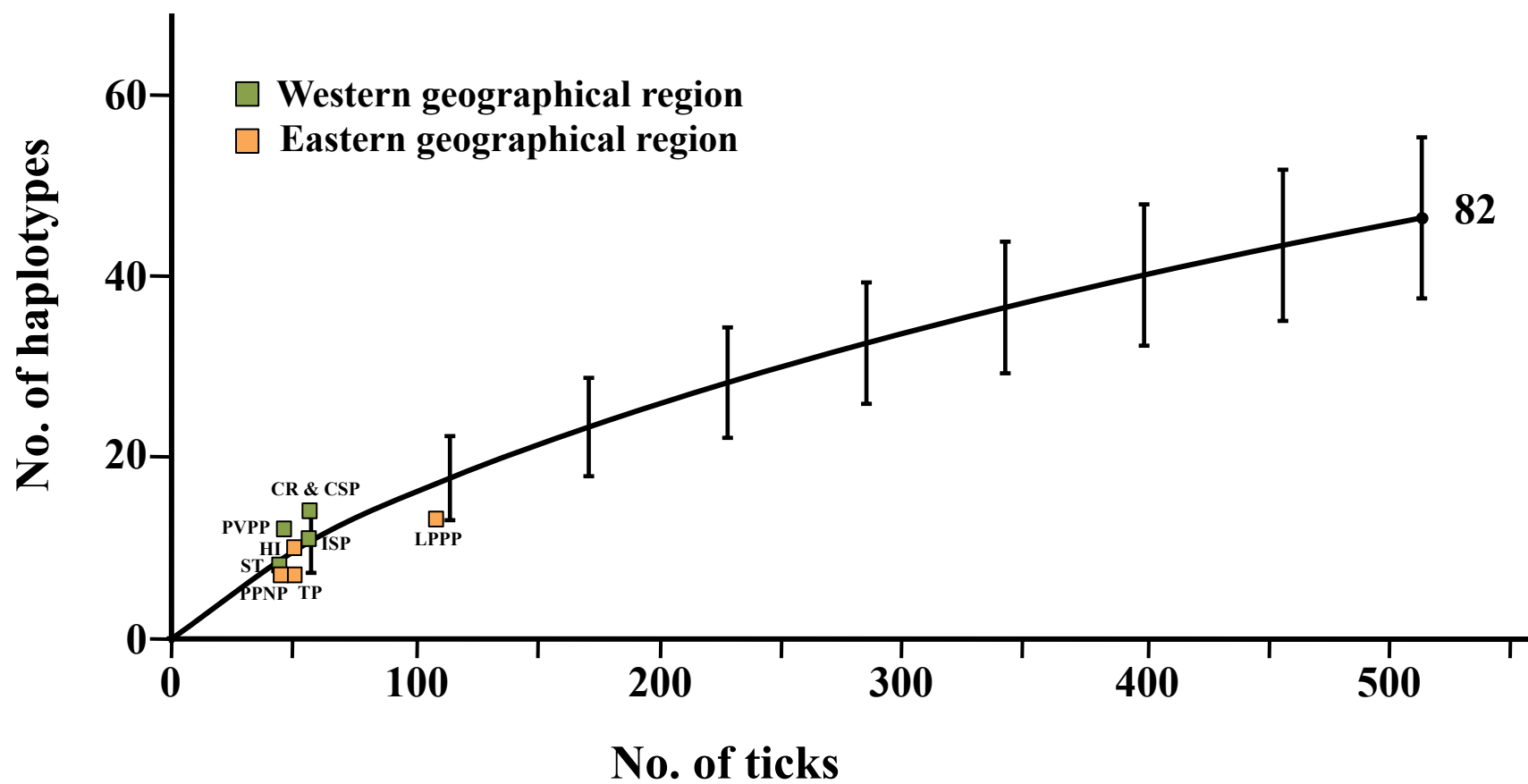

Supplement: Additional file 4: Figure S2. — Rarefaction curve (with 95% confidence intervals) of the haplotype diversity for the I. scapularis populations sampled in the present study. The number next to the curve indicates the total estimated number of haplotypes using the non-parametric Chao 2 estimator. See list of abbreviations for the complete names of localities of the tick populations. [file 13071_2014_530_MOESM4_ESM.pdf]
