# Supplementary material for: Genetic variation in the mitochondrial 16S ribosomal RNA gene of Ixodes scapularis (Acari: Ixodidae)
Source: Parasit Vectors. 2014 Nov 28;7:530. doi: 10.1186/s13071-014-0530-6 (PMC4258262; doi:10.1186/s13071-014-0530-6)
Supplement: Additional file 5: Table S3. — The number of I. scapularis of the different mt 16S rRNA gene haplotypes collected from different geographical regions. [file 13071_2014_530_MOESM5_ESM.docx]

**Table S3** **The number of *I. scapularis* of the different mt 16S rRNA gene haplotypes collected from different geographical regions**

_____________________________________________________________________________________________

Haplotype No. individuals from:

Prairie Midwest Central Northeast Atlantic Total

Canada United States Canada United States Canada

_____________________________________________________________________________________________

Is–1 49 100 85 41 7 282

Is–2 6 2 3 12 2 25

Is–3 0 0 1 0 0 1

Is–4 15 12 5 9 3 44

Is–5 0 0 0 3 0 3

Is–6 1 5 5 1 2 14

Is–7 0 0 6 24 3 33

Is–8 1 2 0 0 0 3

Is–9 4 3 4 0 0 11

Is–10 0 0 2 0 0 2

Is–12 0 0 0 2 0 2

Is–13 3 7 14 0 0 24

Is–14 0 3 0 0 0 3

Is–15 0 0 27 0 0 27

Is–17 0 1 0 0 0 1

Is–20 0 0 3 0 2 5

Is–21 0 0 1 0 0 1

Is–23 1 1 0 0 0 2

Is–24 0 0 1 0 1 2

Is–30 2 0 0 0 0 2

Is–48 0 2 0 0 0 2

Is–49 0 0 0 1 0 1

Is–50 0 0 3 0 0 3

Is–51 3 1 0 0 0 4

Is–52 2 2 0 0 0 4

Is–53 1 1 0 0 0 2

Is–54 1 2 0 0 0 3

Is–55 0 9 0 0 0 9

Is–56 0 2 0 0 0 2

Is–57 5 1 0 0 0 6

Is–58 1 0 0 0 0 1

Is–59 0 2 0 0 0 2

Is–60 0 2 0 0 0 2

Is–61 0 1 0 0 0 1

Is–62 0 4 0 0 0 4

Is–63 1 0 13 5 1 20

Is–64 0 1 0 0 0 1

Is–65 0 1 0 0 0 1

Is–66 0 1 0 0 0 1

Is–67 1 0 0 0 0 1

Is–68 1 0 0 0 0 1

Is–69 3 0 0 0 0 3

Is–70 2 0 0 0 0 2

Is–71 1 0 0 0 0 1

Is–72 0 0 2 0 0 2

Is–73 0 0 2 0 0 2

Is–74 0 0 6 0 0 6

Is–75 0 0 1 0 0 1

Is–76 0 0 3 0 0 3

Is–77 0 0 0 1 1 2

Is–78 0 0 1 0 0 1

Is–79 0 0 0 1 0 1

Total 104 168 188 100 22 582

_____________________________________________________________________________________________
